# Tea at Half Price

**Published:** 1855-03

**Authors:** 


Tea at Half Price.—Laysel, a French Chemist, asserts that if tea is ground like coffee, before hot water is poured upon it, it will yield nearly double the amount of its exhilarating qualities.

If this apparently simple discovery is true, the result of an industrious, but scientific physician, then tea is in effect reduced in price one half, saving to the people of the United States millions of dollars every year. It is certainly worth repeated trial and observation.

